# Patients’ anesthesia preferences for Cesarean delivery: exploring the role of personality beliefs in spinal vs. General anesthesia

**DOI:** 10.1186/s12871-025-03185-w

**Published:** 2025-07-01

**Authors:** Esra Turunc, Sezgin Bilgin, Sevda Akdeniz, Ozgur Komurcu, Leman Tomak, Yasemin Burcu Ustun, Ersin Koksal, Burhan Dost

**Affiliations:** 1 Department of Anesthesiology and Reanimation, Ondokuz Mayis University Faculty of Medicine, Samsun, Turkey; 2 Department of Anesthesiology and Reanimation, Samsun University Faculty of Medicine, Samsun Training and Research Hospital, Samsun, Turkey; 3 Department of Biostatistics and Medical Informatics, Ondokuz Mayis University Faculty of Medicine, Samsun, Turkey

**Keywords:** Anesthesia preference, Cesarean delivery, Spielberger state-trait anxiety inventory (STAI I-II), Personality belief questionnaire short form (PBQ-SF)

## Abstract

**Background:**

This study aimed to compare the personality belief scores of patients who chose either general or spinal anesthesia during cesarean delivery (CD) and explore the relationship between personality beliefs, and anxiety levels.

**Methods:**

This prospective study included expecting mothers, aged 18–45 years, who were classified as ASA II-III, scheduled for elective CD. Anesthesia (general or spinal) was selected based on the patient’s preference. Patients with emergency CD indications, contraindications to either anesthesia type, or inability to complete the evaluation scales were excluded. After collecting sociodemographic data and medical history, patients completed the Spielberger Trait Anxiety Inventory (STAI II) and Personality Belief Questionnaire Short Form (PBQ-SF). On the day of surgery, patients were asked to complete the Spielberger State Anxiety Inventory (STAI I) in the preoperative waiting room. After discharge, the collected results were analyzed and compared based on the women’s anesthetic choices (general or spinal anesthesia).

**Results:**

The study included 150 patients, with 71 expressing a preference for spinal anesthesia and 79 indicating a preference for general anesthesia. The dependent, narcissistic, and borderline personality belief scores, as well as the state anxiety (STAI I) scores, were significantly higher in the spinal anesthesia group than in the general anesthesia group (dependent *p* = 0.003, narcissistic *p* = 0.013, borderline = 0.018, STAI I = 0.01). These differences had small to medium effect sizes (η² = 0.053, 0.040, 0.034, respectively). Spearman’s correlation analysis revealed weak positive correlations between state anxiety (STAI I) scores and dependent (rho = 0.237, *p* = 0.004), narcissistic (rho = 0.287, *p* < 0.001), histrionic (rho = 0.300, *p* < 0.001), and borderline (rho = 0.231, *p* = 0.005) personality belief scores.A weak positive correlation was also observed between trait anxiety (STAI II) scores and dependent personality belief scores (rho = 0.193, *p* = 0.018).

**Conclusions:**

Women who preferred spinal anesthesia had higher scores in dependent, narcissistic, and borderline personality beliefs compared to those who preferred general anesthesia. Although weak, these personality beliefs were also associated with preoperative anxiety. Although these findings are inconclusive, they indicate that personality traits may influence anesthesia preference for CD.

**Trial registration:**

The study was registered on ClinicalTrials.gov (Identifier: NCT06795321).

**Supplementary Information:**

The online version contains supplementary material available at 10.1186/s12871-025-03185-w.

## Introduction

Cesarean deliveries (CD) under general anesthesia continue to be practiced in many countries, although at varying frequencies. In the USA, the overall rate of CD under general anesthesia is 5.8% [1]. In Europe, different rates have been reported for the use of general anesthesia in elective CD (10% in Germany, 30% in Spain, and 34% in the Czech Republic) [2]. In our country, as of 2022, the proportion of CD among all births has been reported as 62.8%. Although the exact rate of those performed under general anesthesia is not known, it is considered to be relatively high [3]. In our hospital, however, according to data obtained from our electronic records, the proportion of CD performed under general anesthesia, including emergency cases, in 2024 was observed to be 56.4%.

Clinical guidelines recommend neuraxial anesthesia as the first choice unless specific contraindications exist [4]. The preference for neuraxial anesthesia is due to the increased risks associated with general anesthesia, such as airway complications, aspiration risk, more significant blood loss, and potential for intraoperative awareness [5]. General anesthesia may be a reasonable option in circumstances where spinal anesthesia is contraindicated or in cases of severe fetal bradycardia, uterine rupture, significant hemorrhage, and placental abruption [4]. One of the reasons for choosing general anesthesia is the patient’s preference. While the preference for general anesthesia varies [6, 7], a small proportion of patients unequivocally reject regional anesthesia [8]. Using general anesthesia for CD is associated with an increased risk of anesthesia-related complications, surgical site infections, and venous thromboembolism [9]. Hence, the unnecessary use of general anesthesia must be minimized. A thorough understanding of the factors influencing patients’ anesthetic preferences may reduce unnecessary general anesthesia by increasing the uptake of neuraxial anesthesia among expecting mothers.

The most frequently reported reasons by patients who refuse regional anesthesia include fear of spinal injury, concerns about pain at the puncture site, and anxiety about witnessing events in the operating room. For those who prefer regional anesthesia, the most influential factors are the fear of not waking up from general anesthesia and the desire to remain awake during childbirth [10]. Research also indicates that anxiety is associated with the choice of anesthetic modality. Previous studies investigating the role of anxiety in CD patients’ anesthesia preferences have yielded conflicting results [11, 12]. Our study hypothesized that patients’ anesthesia preference during CD would differ in certain personality traits. While no studies have been conducted on the relationship between personality traits and anesthesia choice during CD, two previous studies have reported on the relationship between personality traits and maternal delivery preference (CD vs. vaginal delivery). One of these studies reported significantly higher scores in several personality belief domains—including dependent, passive-aggressive, obsessive-compulsive, antisocial, narcissistic, and borderline—in women who preferred CD [13, 14].

The primary aim of this study was to compare the personality belief scores of expecting mothers who experienced a CD under their preferred anesthesia type (general vs. spinal anesthesia). We also aimed to explore the relationship between personality beliefs, anxiety levels, and patient satisfaction.

## Methods

This prospective, single-center, observational study was reported in accordance with the Strengthening the Reporting of Observational Studies in Epidemiology (STROBE) guidelines [15]. Ethical approval was obtained from the Ondokuz Mayıs University Clinical Research Ethics Committee (approval date: 18 April 2022, approval number: 2022/147). The study was carried out between January 2023 and December 2023, in compliance with the principles of the Declaration of Helsinki [16]. The study is registered on ClinicalTrials.gov (Identifier: NCT06795321).

### Study population

Patients, aged between 18 and 45 years, and classified as ASA II or III, who were admitted to the Gynecology and Obstetrics Clinic of Ondokuz Mayıs University Hospital and scheduled for elective CD were included in the study. The anesthesia method (general or spinal anesthesia) was applied at the patient’s discretion. Patients who did not agree to participate in the study, those requiring emergency CD those with an absolute contraindication to general or regional anesthesia, and those lacking educational or mental capacity to complete the evaluation scales, were excluded. Patients were informed about the study during the preoperative visit, the day before the surgery, and written consent was obtained from those who wishes to participate. The advantages and disadvantages of general and spinal anesthesia were explained, and patients were asked to indicate their preference.

#### Instruments

After patients reported their anesthesia preference, we collected sociodemographic data and medical histories, then asked them to complete the Trait Anxiety Inventory (STAI II) and Personality Belief Questionnaire Short Form (PBQ-SF). On the day of surgery, we asked patients to complete the State Anxiety Inventory (STAI I) in the preoperative waiting room (Supplement 1).

The sociodemographic data form included questions about the patients’ name, surname, age, education level, employment status, income level, previous pregnancy and anesthesia experiences, smoking habits, history of psychiatric illness, medication use, and factors influencing their anesthesia preference.

STAI is a 40-item self-report questionnaire designed to measure and distinguish between state anxiety and trait anxiety. It consists of two 20-item subscales, each scored on a 4-point scale, with scores ranging from 20 to 80. State anxiety explores how the person feels at that moment and is measured on a 4-point scale from “not at all” to “very much so.” Trait anxiety refers to how people generally feel. Again, a 4-point scale is used, ranging from “almost never” to “almost always.” The inventory was adapted for Turkish in 1985, with higher scores indicating higher levels of anxiety [17, 18].

The Personality Belief Questionnaire Short FormThe Personality Belief Questionnaire Short Form (PBQ-SF) is a 65-item self-reported questionnaire designed to assess an individual’s personality. The PBQ-SF was created by selecting the seven highest-scoring items for each personality trait from the original PBQ [19]. The form contained statements related to ten different personality disorders/features (avoidant, dependent, passive-aggressive, obsessive-compulsive, antisocial, narcissistic, histrionic, schizoid, paranoid, and borderline). The patients were asked to rate the extent to which they agreed with these statements on a 0–4 scale (0 = not at all, 1 = slightly agree, 2 = moderately agree, 3 = very much agree, and 4 = totally agree). Personality traits were examined based on the scores generated by the responses. The PBQ-SF has been translated into Turkish and its validity and reliability have been confirmed by Bilge et al. [20].

Before discharge, the patient were asked to evaluate their satisfaction with their anesthesia experience using a 4-point Likert scale (1 = poor, 2 = average, 3 = good, and 4 = very good).

#### Outcomes

After discharge, all collected data were analyzed. The results were compared based on the patient’s anesthesia preference (those preferring general anesthesia were analyzed as Group G, and those preferring spinal anesthesia were analyzed as Group S. The primary outcome of the study was the personality belief scores of the patient groups who preferred general or spinal anesthesia for anesthesia management. Secondly, sociodemographic data and post-anesthesia satisfaction scores were compared. Finally, the relationships between personality beliefs, anxiety, and satisfaction scores were investigated.

### Sample size calculation and statistical analysis

Sample size calculation was performed using the subscales of a similar study [13]. The calculation revealed that sample sizes of n1 = 66 and n2 = 66 were coded with 85% power at a 95% confidence interval to detect a 2.6-unit difference with a 4.93 standard deviation between the two groups. Considering the potential data loss, 80 patients were included in each group.

Statistical analyses were performed using SPSS Statistics for Windows, version 21.0 (IBM Corp., Armonk, NY, USA). Data are presented as mean ± standard deviation (SD), median (min-max), and frequency. The Shapiro-Wilk test was used to analyze the normal distribution assumption of the quantitative outcomes. Data were analyzed using the Student’s t-test and Mann–Whitney U test for normally and non-normally distributed data, respectively. Considering that there were 10 subscales for personality belief scores, multivariate analysis of variance (MANOVA) was first performed, followed by univariate analysis of variance (ANOVA) to examine differences in anesthesia preferences. The frequencies were compared using Pearson’s Chi-square and Fisher’s exact tests. Relationships between variables were assessed using Spearman’s rank correlation for non-normal data. Spearman correlation coefficients (rho) were interpreted using the following thresholds: 0.00–0.19 = negligible, 0.20–0.39 = weak, 0.40–0.59 = moderate, 0.60–0.79 = strong, and 0.80–1.00 = very strong correlation [21]. Statistical significance was set at *p* < 0.05.

## Results

**Fig. 1:**
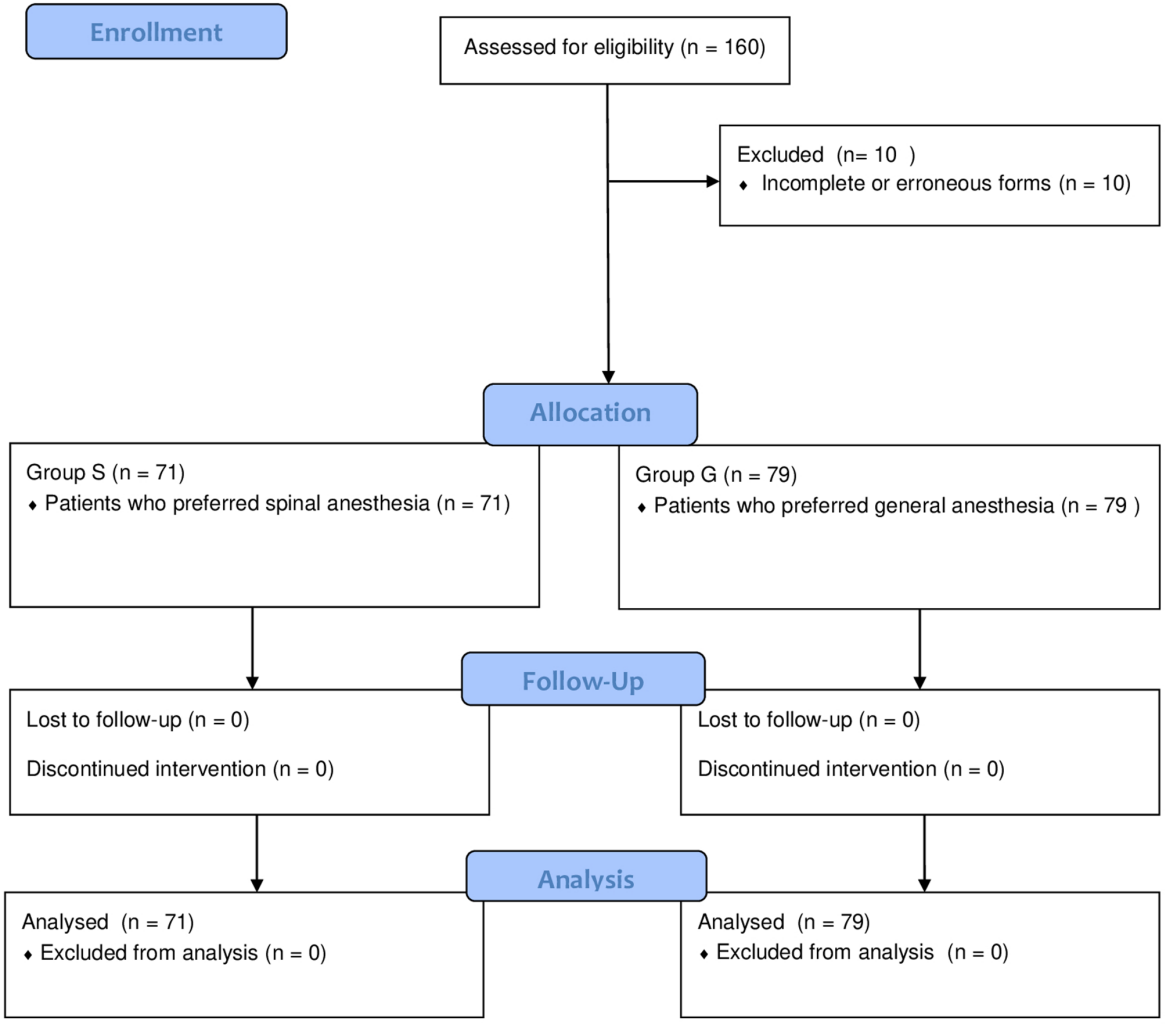
Flow diagram of the study

One hundred sixty patients scheduled for CD were screened for participation in the study. Ten patients were excluded due to incomplete or erroneous forms, resulting in a final sample of 150 patients with analyzable data across both groups. Patients were allocated into two groups according to their preferences for anesthesia management, with 71 patients in Group S and 79 patients in Group G. Figure 1 shows the flow diagram of our study. No significant differences were observed between the groups in terms of age, Body mass index, or other demographic characteristics. (Table 1).

**Table 1:** Comparison of sociodemographic data according to anesthesia preferences

Variable		Group S (*n* = 71)	Group G (*n* = 79)	*p* Value
**Age (year)**		29.15 ± 5.39	30.10 ± 5.75	0.302^1^
**BMI (kg/m**^**2**^ )		31 (21–56)	30.8 (21–38)	0.665^2^
**Education level** ***n (%)***	Elementary school	12 (16.9)	4 (5.1)	0.106^3^
	Middle school	25 (35.2)	27 (34.2)	
	High school graduate	20 (28.2)	27 (34.2)	
	University graduate	14 (19.7)	21 (26.6)	
**Working Status** ***n (%)***	Employed	11 (15.5)	17 (21.5)	0.462^3^
	Not working	60 (84.5)	62 (78.5)	
**Income level** ***n (%)***	Poor	17 (23.9)	15 (19.0)	0.648^3^
	Middle	46 (64.8)	52 (65.8)	
	Good	8 (11.3)	12 (15.2)	
**Psychiatric admission history** ***n (%)***	No	57 (80.3)	71 (89.9)	0.154^3^
	Yes	14 (19.7)	8 (10.1)	
**Psychiatric medication use** ***n (%)***	No	60 (84.5)	72 (91.1)	0.319^3^
	Yes	11 (15.5)	7 (8.9)	
**Smoking status** ***n (%)***	No	53 (74.69	59 (74.7)	0.996^3^
	Yes	18 (25.49	20 (25.3)	
**Number of previous pregnancies** ***n (%)***	1	21 (29.6)	28 (35.4)	0.809^3^
	2	25 (35.2)	21 (26.6)	
	3	16 (22.5)	21 (26.6)	
	≥ 4	9 (12.7)	9 (11.4)	
**Infertility treatment history** ***n (%)***	No	62 (87.3)	75 (94.9)	0.173^3^
	Yes	9 (12.7)	4 (5.1)	
**Previous delivery method** ***n (%)***	Vaginal delivery	8 (11.3)	15 (19.0)	0.201^3^
	C-section	43 (60.6)	37 (46.8)	
	None	20 (28.2)	27 (34.2)	
**Type of anesthesia in previous CD** ***n (%)***	Regional	37 (52.1)	19 (24.1)	**0.002** ^3*^
	General	6 (8.5)	18 (22.8)	
	N/A	28 (39.4)	42 (53.2)	
**Previous anesthesia history** ***n (%)***	No	52 (73.2)	68 (86.1)	0.079^3^
	Yes	19 (26.8)	11 (13.9)	
**Advers events** ***n (%)***	No	67 (94.4)	72 (91.1)	0.658^3^
	Yes	4 (5.6)	7 (8.9)	
**What influenced the choice of anesthesia?** ***n (%)***	Doctor (anesthetist or gynecologist)	61 (88.4)	39 (52.7)	**< 0.001** ^**3**^
	Media	2 (2.9)	13 (17.6)	
	Relatives	6 (8.7)	22 (29.7)	

Statistically significant difference is highlighted in bold

*Abbreviations: ASA* American Society of Anesthesiologists, *BMI* Body Mass Index, *CD* Cesarean delivery

1: Independent samples t test

2: Mann_Whitney U Test

3: Chi square tests

Regarding medical history, 52.1% of the patients in Group S had undergone spinal anesthesia during their previous CD. Meanwhile, 53.2% of the patients in the general anesthesia group had no prior CD experience. The two groups differed significantly in their previous history of CD (*p* < 0.002). Other medical histories were not significantly different between the groups. Regarding the question, ‘What influenced your anesthesia preference?‘, 88.4% of those in Group S and 52.5% of those in Group G indicated that their decision was influenced by their doctors. A significant difference was found between the groups regarding factors influencing the choice of anesthesia (*p* < 0.001). Table 1 shows the sociodemographic data, medical history, and the factors influencing anesthesia choice, between the groups.

When comparing personal belief scores according to anesthesia preferences, the overall multivariate analysis of variance (MANOVA) on the ipsative subscale scores of the 10 PBQ-SF subscales was significant (mult F = 2.79, *p* = 0.004). In the subgroup comparisons among anesthesia types, statistical differences were observed for the dependent, narcissistic, and borderline subgroups; the p-values were 0.005, 0.014, and 0.023, respectively. The effect sizes of these differences ranged from small to medium (η2, 0.053, 0.040, 0.034, respectively) (Table 2). When comparing anxiety, and satisfaction scores according to anesthesia preference, it was found that the state anxiety (STAI I) scores were significantly higher in Group S than in Group G (*p* = 0.01) (Table 3).

**Table 2:** Comparison of personality belief questionnaire short form scores according to anesthesia preferences

	Group S (*n* = 71)	Group G (*n* = 79)	F	*p*	η2
	Mean ± SD	Mean ± SD			
Avoidant	15.17 ± 4.44	14.51 ± 3.95	0.94	0.335	0.006
Dependent	12.62 ± 5.65	9.92 ± 5.80	8.28	0.005	0.053
Passive-Aggressive	12.93 ± 4.56	12.42 ± 4.56	0.47	0.494	0.003
Obsessive-Compulsive	13.68 ± 4.42	13.81 ± 3.85	0.04	0.843	0.000
Antisocial	11.17 ± 6.09	10.76 ± 5.04	0.20	0.653	0.001
Narcissistic	12.23 ± 5.44	9.99 ± 5.55	6.20	** 0.014**	0.040
Histrionic	10.83 ± 5.40	9.24 ± 5.60	3.12	0.079	0.021
Schizoid	14.07 ± 4.56	14.06 ± 3.96	0.00	0.992	0.000
Paranoid	14.24 ± 5.03	12.95 ± 5.07	2.44	0.121	0.016
Borderline	12.01 ± 5.30	9.96 ± 5.60	5.28	** 0.023**	0.034

One-way ANOVA; η² = effect size. Statistically significant difference is highlighted in bold

**Table 3:** Comparison of STAI I, STAI II and satisfaction scores according to anesthesia preferences

	Group S (*n* = 71) Median (min-max)	Group G (*n* = 79) Median (min-max)	*p* Value
**STAI I**	40 (24–55)	36 (26–54)	**0.01**
**STAI II**	47 (28–62)	45 (27–65)	0.057
**Post-anesthesia satisfaction score**	4 (2–4)	3 (2–4)	0.203

Mann Whitney U test, Statistically significant difference is highlighted in bold

*Abbreviations: STAI II*, Spielberger Trait Anxiety Inventory, *STAI I* Spielberger State Anxiety Inventory

Spearman’s correlation analysis was used to assess the relationship between personality belief, anxiety, and satisfaction scores. The analysis showed a weak positive correlation between state anxiety scores (STAI I) and dependent (rho = 0.237, *p* = 0.004), narcissistic (rho = 0.287, *p* < 0.001), histrionic (rho = 0.300, *p* < 0.001), and borderline (rho = 0.231, *p* = 0.005) personality belief scores. The correlations between STAI I and passive-aggressive (rho = 0.161, *p* = 0.049), antisocial (rho = 0.185, *p* = 0.024) personality belief scores were negligible.

A negligible positive correlation was observed between trait anxiety scores (STAI II) and dependent (rho = 0.193, *p* = 0.018) personality belief scores, whereas a negligible negative correlation was found between trait anxiety scores (STAI II) and schizoid (rho=−0.185, *p* = 0.023) personality belief scores. In the correlation analysis between post-anesthesia satisfaction and personality belief scores, a negligible negative correlation was found between antisocial (rho=−0.184, *p* = 0.025) personality beliefs and postoperative satisfaction scores (Table 4).

**Table 4:** Correlations between STAI I, STAI II and anesthesia satisfaction scores and PBQ scores

Spearman correlation	STAI I		STAI II		Post-anesthesia satisfaction	
	Rho	*p*	Rho	*p*	Rho	*p*
Avoidant	0.002	0.984	0.078	0.340	−0.005	0.950
Dependent	**0.237**	**0.004**	**0.193**	**0.018**	−0.075	0.361
Passive-Aggressive	**0.161**	**0.049**	−0.021	0.795	−0.084	0.308
Obsessive Compulsive	0.136	0.097	−0.038	0.643	−0.138	0.091
Antisocial	**0.185**	**0.024**	−0.037	0.657	**−0.184**	**0.025**
Narcissistic	**0.287**	**< 0.001**	0.018	0.830	−0.125	0.127
Histrionic	**0.300**	**< 0.001**	0.079	0.339	−0.133	0.104
Schizoid	0.157	0.055	**−0.185**	**0.023**	−0.009	0.915
Paranoid	0.100	0.223	−0.030	0.713	−0.120	0.143
Borderline	**0.231**	**0.005**	0.149	0.068	−0.131	0.109

Spearman Correlations, Statistically significant difference is highlighted in bold

*Abbreviations: STAI II*, Spielberger Trait Anxiety Inventory, *PBQ-SF*, Personality Belief Questionnaire Short Form, *STAI I* Spielberger State Anxiety Inventory

## Discussion

The study results revealed that patients who preferred spinal anesthesia had higher scores on dependent, narcissistic, and borderline personality beliefs. Although these differences were statistically significant, the effect sizes were in the small to approaching moderate range, and thus should be interpreted with caution. In addition, patients in the spinal anesthesia group also exhibited higher state anxiety scores compared to those who preferred general anesthesia. More than half of the patients who preferred spinal anesthesia had previously undergone the same method during a prior CD, whereas more than half of those who preferred general anesthesia had no prior CD experience. Importantly, 88.4% of the patients in the spinal anesthesia group and 52.7% in the general anesthesia group reported that their decision was influenced by their doctors.

Personality beliefs are constructs that shape specific behavioral patterns. Individuals may hold some of these beliefs at the nonclinical or nonpathological level. However, in clinically significant personality disorders, these behaviors become more pronounced as manifestations of underlying cognitive frameworks [22]. Among our findings, the most notable was the higher dependent personality belief scores observed in patients who preferred spinal anesthesia. Although the effect size was small, this difference reached statistical significance and showed a weak correlation with state anxiety (rho = 0.237, *p* = 0.004), and a negligible correlation with trait anxiety (rho = 0.193, *p* = 0.018). High levels of dependent personality beliefs can cause individuals, particularly women, to perceive themselves as helpless, vulnerable, and in need of assistance. In a study investigating the relationship between personality traits and women’s choice of delivery method, dependent personality scores were found to be higher in the group that chose CD [10]. Additionally, difficulty in expressing disagreements with others and the fear of losing support or approval are behavioral criteria for dependent personality disorder [11]. In such individuals, the high need for care results in separation anxiety, submissive behavior, and an attachment-seeking attitude [23]. In the spinal anesthesia group, these features may have manifested as a desire to stay awake during the surgery or a fear that the anesthesiologist would leave the room if they were to undergo general anesthesia. Dependent personality disorder is classified as Cluster C disorders, also referred to as anxious/fearful disorders [12]. It is also known to be a risk factor for mood disorders [24] and anxiety disorders [25]. The association between dependent personality beliefs and both state and trait anxiety in our study aligns with available evidence from the literature.

It is not uncommon for individuals to simultaneously exhibit personality traits from different clusters [23]. Dependent, histrionic, and borderline personality traits are more frequently observed in women [26]. In our study, borderline and narcissistic personality beliefs also scored higher in the spinal anesthesia group. Borderline and narcissistic personality disorders are classified within Cluster B, characterized by dramatic-emotional disorders [23]. Individuals with borderline personality beliefs view themselves as vulnerable, deprived, and powerless. As a result, they experience fear of abandonment and struggle in interpersonal relationships [27]. Narcissistic personality beliefs, on the other hand, manifest as a sense of being out of control, unique, superior, and above the rules. Individuals with this disorder tend to have high expectations of others. A recent study found a negative correlation between narcissistic personality traits and subjective well-being and coping abilities [28]. Similarly, another study found a negative correlation between neuroticism, which leads to emotional lability, and perceived safety during childbirth [29]. In our study, narcissistic and borderline personality beliefs were weakly correlated with state anxiety, suggesting that increased anxiety may have led patients to desire the presence of someone who could provide a sense of security. Thus, the preference for spinal anesthesia in these patients could be explained by their desire to feel safe, remain in control, and have someone to support them during childbirth.

Another notable finding was that patients who chose spinal anesthesia had higher state anxiety scores than those who chose general anesthesia. The higher personality belief scores found in the spinal anesthesia group and their correlation with state anxiety may have contributed to this result. Contrary to our findings, a study conducted in Pakistan found that patients who chose general anesthesia had higher anxiety levels [12]. In another study, no differences were found in preoperative anxiety between patients who received general or spinal anesthesia [11]. While these studies assessed anxiety using a visual analog scale (VAS), we used the STAI scale. Although one study reported a correlation between the STAI and VAS for measuring anxiety, it included patients scheduled for ear, nose, throat and abdominal surgery [30]. The emotional state of pregnant women may differ from that of other patients, which could account for the conflicting results in anxiety assessments using self-reported scales.

No prominent personality beliefs were identified in the patients who chose general anesthesia. Interestingly, more than half of the patients who underwent general anesthesia also underwent their first CD. In contrast, more than half of the patients in the spinal anesthesia group were already familiar with the method used in the previous CD. This aligns with findings in the literature suggesting that women’s reasons for rejecting spinal anesthesia or choosing general anesthesia are generally non-scientific and often stem from insufficient information. Additionally, studies have shown that patients who undergo spinal anesthesia during a CD tend to choose the same method again [14].

One study reported that anesthesiologists and gynecologists, along with income and education level, were key factors in anesthesia choice [31]. In our study, 88.4% of the patients who preferred spinal anesthesia reported that their decision was influenced by their doctors, suggesting that even without direct persuasion, informing patients may have increased the selection of spinal anesthesia. Given the patients’ lack of information and the influence of physicians, this highlights the importance of providing information about anesthesia options. Recognizing the patients’ personality traits during this information session could also help with identifying their needs, making appropriate recommendations, and potentially reducing unnecessary general anesthesia. We acknowledge that knowing a patient’s personality type may not be sufficient to change their opinion regarding anesthesia choice. However, a better understanding of the potential causes of patients’ concerns could serve as a guide in providing reassurance and tailored communication.

Our study has some limitations. It was conducted in a single tertiary center with a relatively homogeneous patient population, which may limit the generalizability of our findings. Cultural and institutional differences could also influence anesthesia preferences and help explain the variation in general anesthesia rates for CD across countries. The continued use of general anesthesia in CD remains a relevant clinical issue.

Additionally, the influence of the obstetric provider may represent a confounding factor in interpreting the relationship between personality traits and anesthesia choice. However, in our study design, we did not distinguish between anesthesiologists and obstetricians when recording physician influence on anesthetic modality choice, and all patients were provided with standardized, neutral information about both anesthesia methods by the anesthesiology team. This may have helped minimize the impact of this potential confounder.

Finally, our study did not include specific questions aimed at identifying the reasons behind patients’ anesthesia preferences. Collecting such data in future studies may provide further insights into the relationship between personality traits and decision-making in this context.

## Conclusion

The results of this study indicated that patients who preferred spinal anesthesia had higher scores on dependent, narcissistic, and borderline personality beliefs than those who preferred general anesthesia. Although the effect sizes were small, these personality beliefs were weakly associated with preoperative anxiety and may reflect subtle psychological factors influencing anesthesia preference. While these findings are insufficient to draw firm conclusions, they suggest that personality traits may play a role in determining anesthesia preference during CD. Large-scale studies that specifically investigate the reasons behind patient preference for certain anesthesia modalities are needed to better understand this issue. Additionally, our findings regarding the patients’ tendency to choose spinal anesthesia again after a previous experience and the influence of doctors on patients’ decisions, are noteworthy. Overall, these findings emphasize the importance of providing personalized information before elective CD to minimize unnecessary general anesthesia.

## Supplementary Information

Supplementary Material 1.

## Data Availability

The datasets used and/or analysed during the current study available from thr corresponding author on reasonable request.

## Abbreviations

ASA: American Society of Anesthesiologists
CD: Cesarean delivery
VAS: Visual Analog Scale
PBQ-SF: Personality Belief Questionnaire Short Form
STAI-II: Spielberger Trait Anxiety Inventory
STAI-I: Spielberger State Anxiety Inventory
