# Supplementary material for: Patients’ anesthesia preferences for Cesarean delivery: exploring the role of personality beliefs in spinal vs. General anesthesia
Source: BMC Anesthesiol. 2025 Jul 1;25:313. doi: 10.1186/s12871-025-03185-w (PMC12210962; doi:10.1186/s12871-025-03185-w)
Supplement: Supplementary file 1 — Supplementary Material 1. [file 12871_2025_3185_MOESM1_ESM.pdf]

Questionnaire Form

Date:

Protocol No:

Initials (Name and Surname):

Age:

Height:

Weight:

Preferred Type of Anesthesia:

☐ Regional Anesthesia ( )

☐ General Anesthesia ( )

Education Level:

☐ Literate ( )

☐ Primary School ( )

☐ Middle School ( )

☐ High School Graduate ( )

☐ University Graduate ( )

Employment Status / Occupation:

☐ Employed ( )

☐ Unemployed ( )

Have You Previously Consulted a Psychiatrist?

☐ Yes ( )

☐ No ( )

Are You Currently Using Psychiatric Medication?

☐ Yes ( )

☐ No ( )

Do You Have a Family History of Mental Illness?

☐ Yes ( )

☐ No ( )

Do You Smoke

□ Yes ( )

□ No ( )

Current Pregnancy Number:

Have You Received Infertility Treatment?

□ Yes ( )

□ No ( )

Previous Delivery Method:

If Cesarean, Type of Anesthesia Used:

□ Regional ( )

□ General ( )

Previous Anesthesia Experience for Other Reasons:

□ Yes ( )

□ No ( )

Have You Experienced Any Adverse Outcomes During Anesthesia?

□ Yes ( )

□ No ( )

Factors Influencing Your Choice of Anesthesia:

□ Obstetrician ( )

□ Anesthesiologist ( )

□ Television ( )

□ Internet ( )

□ Book ( )

□ Relatives ( )

Father’s Age:

Father’s Education Level:

□ Literate ( )

□ Primary School ( )

□ Middle School ( )

□ High School Graduate ( )

□ University Graduate ( )

Father’s Employment:

□ Employed ( )

□ Unemployed ( )

Parental Relationship:

□ Living together ( )

□ Divorced ( )

□ If divorced, how old were you at the time?

Number of Siblings:

□ Sisters: __

□ Brothers: __

□ You are the __ child

Primary Caregiver (0–6 years):

□Parents

□ Relatives

□Institution

Are you currently living with your family?

□ Yes ( )

□ No ( )

Has your family experienced any major migration/separation?

□ Yes ( )

□ No ( )

If yes, how old were you? ……….

Have you ever consulted a psychiatrist?

□ Yes ( )

□ No ( )

Any family history of psychiatric disorders?

□ Yes ( )

□ No ( )

If yes, what is the relationship?

□ Mother / Father / Sibling ( )

□ Maternal or Paternal Grandparents ( )

Do you smoke?

□ Yes ( )

□ No ( )

If yes, how much?

__ cigarettes/day

For how long?

□ __ days / __ weeks / __ years

Do you consume alcohol?

□ Yes ( )

□ No ( )

If yes, how much?

□__ units/day

For how long?

__ days / __ weeks / __ years

Have you ever used substances (e.g., cannabis, heroin, cocaine, pills, thinner)?

 Yes ( )

 No ( )

Have you ever attempted suicide?

Yes ( )

No ( )

If yes, how many times? ………….

STAI FORM TX – I

INSTRUCTIONS:
Below are a number of statements that people use to describe their feelings. Please read each statement and indicate how you feel right now, at this moment, by selecting the appropriate response from the options on the right. There are no right or wrong answers. Do not spend too much time on any one statement; choose the answer that best reflects your immediate feeling.

		**Not at all**	**A little**	**Quite a bit**	**Very much so**
1.	I feel calm	(1)	(2)	(3)	(4)
2.	I feel secure	(1)	(2)	(3)	(4)
3	I am tense	(1)	(2)	(3)	(4)
4	I feel regretful	(1)	(2)	(3)	(4)
5.	I feel at ease	(1)	(2)	(3)	(4)
6	I feel upset	(1)	(2)	(3)	(4)
7	I am worried about possible misfortunes	(1)	(2)	(3)	(4)
8.	I feel rested	(1)	(2)	(3)	(4)
9	I feel anxious	(1)	(2)	(3)	(4)
10.	I feel comfortable	(1)	(2)	(3)	(4)
11.	I feel self-confident	(1)	(2)	(3)	(4)
12	I feel annoyed	(1)	(2)	(3)	(4)
13	I feel very nervous	(1)	(2)	(3)	(4)
14	I feel highly strung	(1)	(2)	(3)	(4)
15.	I feel relaxed	(1)	(2)	(3)	(4)
16.	I am satisfied with myself	(1)	(2)	(3)	(4)
17	I feel worried	(1)	(2)	(3)	(4)
18	I feel confused from excitement	(1)	(2)	(3)	(4)
19.	I feel happy	(1)	(2)	(3)	(4)
20.	I feel cheerful	(1)	(2)	(3)	(4)

STAI FORM TX – II

INSTRUCTIONS:
Below are a number of statements that people use to describe their feelings. Please read each statement and indicate how you feel right now, at this moment, by selecting the appropriate response from the options on the right. There are no right or wrong answers. Do not spend too much time on any one statement; choose the answer that best reflects your immediate feeling.

		Almost never	Sometimes	Often	Almost always
21.	I generally feel cheerful	(1)	(2)	(3)	(4)
22	I usually get tired easily	(1)	(2)	(3)	(4)
23	I often cry easily	(1)	(2)	(3)	(4)
24	I wish I could be as happy as others	(1)	(2)	(3)	(4)
25	I miss opportunities due to indecisiveness	(1)	(2)	(3)	(4)
26.	I generally feel rested	(1)	(2)	(3)	(4)
27.	I am generally calm and composed	(1)	(2)	(3)	(4)
28	I feel overwhelmed by difficulties	(1)	(2)	(3)	(4)
29	I worry over unimportant matters	(1)	(2)	(3)	(4)
30.	I am usually happy	(1)	(2)	(3)	(4)
31	I take things seriously and worry	(1)	(2)	(3)	(4)
32	I usually lack self-confidence	(1)	(2)	(3)	(4)
33.	I generally feel secure	(1)	(2)	(3)	(4)
34	I avoid difficult situations	(1)	(2)	(3)	(4)
35	I often feel sad	(1)	(2)	(3)	(4)
36.	I am generally satisfied with life	(1)	(2)	(3)	(4)
37	I am disturbed by random thoughts	(1)	(2)	(3)	(4)
38	I take disappointments so seriously that I can't forget them	(1)	(2)	(3)	(4)
39.	I am a stable and determined person	(1)	(2)	(3)	(4)
40	Recent worries have been disturbing me	(1)	(2)	(3)	(4)

PBQ-S1

Personality Belief Questionnaire – Short

[illegible]

Please read the statements below and indicate HOW MUCH YOU BELIEVE EACH ONE. Decide based on how you feel about each statement MOST OF THE TIME.

**4**	**3**	**2**	**1**	**0**
Totally Believe	Strongly Believe	Moderately Believe	Slightly Believe	Do Not Believe at All

HOW MUCH DO YOU BELIEVE IT?

	**4**	**3**	**2**	**1**	**0**
1. Experiencing humiliation or inadequacy is intolerable.	4	3	2	1	0
2. I must avoid distressing situations at all costs.	4	3	2	1	0
3. If people act friendly, they may be trying to use or exploit me.	4	3	2	1	0
4. I must resist the control of authorities, yet also seek their approval.	4	3	2	1	0
5. I cannot tolerate distressing emotions.	4	3	2	1	0
6. Flaws, defects, or mistakes are intolerable.	4	3	2	1	0
7. Other people often demand too much.	4	3	2	1	0
8. I should be the center of attention.	4	3	2	1	0

9. Without a system, everything falls apart.	4	3	2	1	0
10. It is intolerable not to receive the respect or entitlement I deserve.	4	3	2	1	0
11. It is important to do a perfect job in everything.	4	3	2	1	0
12. I prefer doing things alone over doing them with others.	4	3	2	1	0
13. If I'm not careful, people will try to use or control me.	4	3	2	1	0
14. Other people have hidden motives.	4	3	2	1	0
15. The worst possible thing is to be abandoned.	4	3	2	1	0
16. Others should recognize how special I am.	4	3	2	1	0
17. People deliberately put me down.	4	3	2	1	0
18. I need help from others to make decisions or tell me what to do.	4	3	2	1	0
19. Details are extremely important.	4	3	2	1	0
20. If people act bossy, I have the right to ignore them.	4	3	2	1	0
21. Authorities are intrusive, demanding, and controlling.	4	3	2	1	0
22. The way to get what I want is to impress or amuse others.	4	3	2	1	0
23. I must do whatever it takes to get ahead.	4	3	2	1	0

24. If people find things out about me, they will use them against me.	4	3	2	1	0
25. Relationships are messy and interfere with freedom.	4	3	2	1	0
26. Only people as intelligent as I am can understand me.	4	3	2	1	0
27. Because I’m so superior, I deserve special treatment and privileges.	4	3	2	1	0
28. It is important for me to be independent from others.	4	3	2	1	0
29. I often feel better when I’m alone.	4	3	2	1	0
30. Unless I reach the highest standards, everything will fall apart.	4	3	2	1	0
31. Distressing feelings will escalate and get out of control.	4	3	2	1	0
32. We live in a jungle, and only the strong survive.	4	3	2	1	0
33. I should avoid drawing attention and be as inconspicuous as possible.	4	3	2	1	0
34. If I can’t keep people interested in me, they won’t like me.	4	3	2	1	0
35. If I want something, I should do whatever it takes to get it.	4	3	2	1	0
36. It's better to be alone than to be “stuck” with someone.	4	3	2	1	0
37. Unless I impress or entertain people, I’m nothing.	4	3	2	1	0

38. If I don’t take charge first, others will dominate me.	4	3	2	1	0
39. Tension in a relationship means it’s going bad; I should end it	4	3	2	1	0
40. If I don’t perform at the highest level, I’m a failure.	4	3	2	1	0
41. Meeting deadlines, complying with demands, and conformity are a blow to my pride.	4	3	2	1	0
42. I am often treated unfairly and I deserve to get my share by any means.	4	3	2	1	0
43. If people get close, they’ll find out what I’m really like and leave me.	4	3	2	1	0
44. I am needy and weak.	4	3	2	1	0
45. I am helpless when I’m alone.	4	3	2	1	0
46. Others should meet my needs.	4	3	2	1	0
47. If I follow rules as expected, it limits my freedom.	4	3	2	1	0
48. If I give others the chance, they’ll take advantage of me.	4	3	2	1	0
49. I must always be on guard.	4	3	2	1	0
50. My privacy is more important than closeness to others.	4	3	2	1	0
51. Rules are arbitrary and stifling.	4	3	2	1	0
52. It’s awful when people ignore me.	4	3	2	1	0
53. I don’t care what people think of me.	4	3	2	1	0

54. To be happy, I need others to pay attention to me.	4	3	2	1	0
55. If I entertain people, they won't notice my weaknesses.	4	3	2	1	0
56. I need someone around at all times to help me or in case something bad happens.	4	3	2	1	0
57. Any flaw or mistake can lead to disaster.	4	3	2	1	0
58. Because I'm very talented, people should step aside to let me advance.	4	3	2	1	0
59. If I don't push others, they will dominate me.	4	3	2	1	0
60. I don't have to follow rules that apply to other people.	4	3	2	1	0
61. The best way to get things done is by force or cunning.	4	3	2	1	0
62. I must always maintain access to someone.	4	3	2	1	0
63. I'm basically alone—unless I attach myself to a stronger person.	4	3	2	1	0
64. I cannot trust other people.	4	3	2	1	0
65. I'm not as strong as others.	4	3	2	1	0

Postoperative Satisfaction Scale

☐Poor

☐Fair

☐Good

☐Very Good
